# Investigating the determinants of vaccine hesitancy within undergraduate students’ social sphere

**DOI:** 10.1007/s10389-021-01538-6

**Published:** 2021-04-09

**Authors:** Alessandro Siani, Megan Driscoll, Tia-mai Hurst, Tutu Coker, Alice Georgina Grantham, Amrit Bunet

**Affiliations:** School of Biological Sciences, University of Portsmouth, King Henry Building, King Henry 1st Street, Portsmouth, PO1 2DY UK

**Keywords:** Vaccine hesitancy, University, Undergraduate students, Age, Ethnicity, Religion, Qualifications

## Abstract

**Aims:**

Vaccine hesitancy is widely recognised as one of the most serious threats to current global health. While the causes underlying vaccine hesitancy have been extensively described and several mitigation strategies trialled amongst current and prospective parents, there is a relative scarcity of research investigating its extent and causative factors amongst university students, a critical demographic due to its temporal proximity to the average child-rearing age. The present study sought to address this literature gap by elucidating the social and demographic factors that might underpin vaccine hesitancy in university students.

**Subject and methods:**

An anonymous online survey was carried out to investigate the opinions and perspectives on the practice of vaccination within undergraduate students’ social sphere. The statistical significance of the differences observed between groups of participants was analysed using non-parametric tests of variance.

**Results:**

Amongst the 739 volunteers who participated in the survey, vaccine confidence varied significantly (*p* < 0.001) with age, ethnicity and religion, and to a lesser (yet still statistically significant) extent (*p* < 0.05) with graduate status. No statistically significant differences were observed with regard to gender or number of children.

**Conclusions:**

By shedding new light on the factors underpinning vaccine hesitancy within undergraduate students’ social network, the present study provides a stepping stone towards the development of targeted mitigation strategies.

## Introduction

### Historical outline of vaccine hesitancy

Vaccine hesitancy, defined as the delay or refusal of available vaccinations, has been recognised by the World Health Organization (WHO) as one of the ten biggest current threats to global public health, on par with, among others, antimicrobial resistance, air pollution and climate change (World Health Organization 2019). Far from being a recent phenomenon, the opposition to immunisation predates even the development of the first vaccine by Edward Jenner in 1796; several historical sources indicate that the practice of variolation, widely considered as a rudimentary predecessor to vaccination, was often received with vocal opposition upon its introduction in Britain and the United States in the early eighteenth century (DeLacy 2016; Wagstaffe 1722).

Anti-vaccination movements gained significant momentum in the last decades of the twentieth century, as exemplified by the long-lasting impact of the controversy spurred by the publication of Andrew Wakefield’s fraudulent article in *The Lancet*, which suggested a causative link between the measles/mumps/rubella (MMR) vaccine and the insurgence of gastrointestinal and neurological symptoms in children (Godlee et al. 2011; Wakefield et al. 1998). Moreover, the global availability of largely unchecked information made possible by the World Wide Web has facilitated the spread of pseudo-scientific and conspiratorial worldviews, providing fertile ground for the affirmation of anti-vaccination movements (Kata 2010). Coinciding with the rise of nationalistic populism in developed countries, the turn of the twenty-first century saw a further consolidation of anti-intellectual and anti-scientific ideologies, resulting in the decrease of vaccine coverage and consequent re-emergence of vaccine-preventable diseases (VPD) (Siani 2019).

### Causes of vaccine hesitancy

In order to fully understand the nature of vaccine hesitancy and its underlying causes, it is essential to acknowledge that vaccine acceptance is not an “all or nothing” scenario whereby people either accept or refuse all vaccinations unconditionally. Instead, the vaccination decision-making process is influenced by a combination of several intrinsic and extrinsic factors, which ultimately determine the position of each individual or group along the vaccine acceptance spectrum. These underlying factors have been largely investigated and described by the Strategic Advisory Group of Experts (SAGE), a WHO working group instituted to study and tackle vaccine hesitancy. SAGE proposed a framework based on two key models, namely “Complacency, Convenience and Confidence” (3Cs) and the “Determinants of Vaccine Hesitancy Matrix” (World Health Organization 2014). 3Cs, the simpler of the two models, postulates that the causes of vaccine hesitancy can be grouped into three broad categories: Complacency refers to a reduction in perceived VPD risk, whereby some diseases (in most cases due to high vaccine coverage and the effectiveness of the vaccinations themselves) are considered too rare or not serious enough to be worth vaccinating against. Convenience encompasses factors related to the geographical availability of vaccine services in a certain area, and the physical and economic accessibility to them. Confidence is defined as trust in the policymakers who promote the vaccinations, in the healthcare workers (HCW) who administer them and in the vaccines themselves. The “Determinants of Vaccine Hesitancy Matrix”, while less intuitive than the 3Cs model, provides a more detailed account of the complexity of causative factors underpinning vaccine hesitancy. According to the matrix, the determinants of vaccine hesitancy can be subdivided into contextual influences “arising due to historic, socio-cultural, environmental, health system/institutional, economic or political factors”, individual and group influences “arising from personal perception of the vaccine or influences of the social/peer environment” and vaccine-specific issues “directly related to vaccine or vaccination”.

### Tackling vaccine hesitancy

The SAGE models constitute a comprehensive framework for the development of strategies aimed at mitigating the extent and impact of vaccine hesitancy. Meaningful interventions should be designed not only to tackle hesitancy in adults of child-rearing age but, perhaps more importantly, to also provide children and adolescents with adequate scientific and digital literacy throughout all levels of their educational pathway to inform their future decision-making process. Over the last decades, a wealth of intervention strategies have been developed, deployed and evaluated by healthcare and academic institutions around the world. Evidence-based strategies to address vaccine hesitancy in adults can be roughly divided into three main categories, namely individual-level interventions focusing on current or soon-to-be parents, individual-level interventions focusing on supporting and training HCW, and community-level interventions aimed at involving local authority figures and religious leaders (European Centre for Disease Prevention and Control 2017). On the other hand, while several studies have pinpointed existing pedagogical strategies aimed at implementing basic elements of immunology and epidemiology in a level-appropriate fashion across school curricula (Arede et al. 2019), research on this matter remains relatively sparse, particularly with regard to strategies addressing vaccine hesitancy in higher education (HE) students and their social context.

### Aims of the study

The existence of a literature gap with regard to the extent and determinants of vaccine hesitancy in university students should be considered a cause for concern, as it undermines the development and implementation of targeted interventions. This is particularly important considering the temporal proximity between HE years and the critical time window for child-rearing and vaccination decision-making in many prospective parents. As pinpointed by the SAGE matrix, peer influence and the wider social context play a key role in determining an individual’s attitude towards vaccination uptake. With the current study, we sought to investigate perspectives on vaccination within undergraduate students’ social sphere to elucidate which demographic, social and cultural factors are associated with vaccine confidence (as defined in the SAGE 3Cs model) in the selected population.

## Methods

### Survey design

The questionnaire (Table 1) used in this study, partially adapted from the WHO “Determinants of vaccine hesitancy: sample survey questions” question bank, was composed of three main sections. The first section contained six multiple-choice questions designed to collect the demographic characteristics used as independent variables in this study. The second part consisted of twelve Likert-type questions to quantitatively gauge the respondents’ attitudes with regard to vaccination on a scale from 1 (strongly disagree) to 5 (strongly agree). The final section allowed the participants to further elaborate their opinions via five yes/no questions, each followed by a short open-ended question. The Vaccine Confidence Score (VCS) used in this study was calculated by summing the answers to the five Likert-type questions indicated by an asterisk in Table 1. As a five-point Likert scale was used in each question, the VCS ranges from 5 (minimum confidence) to 25 (maximum confidence).

**Table 1:** Questionnaire used in the survey. The questions marked with an asterisk (*) were used in the calculation of the VCS. Note that not all of the questions presented in this table are discussed in this paper

**Section 1: Personal information (multiple-choice questions)**	
What is your age?	
What gender do you identify as?	
How many children do you have?	
What qualifications do you have?	
What is your ethnic group?	
What is your religion/spiritual belief?	
**Section 2: Attitude on vaccinations (5-point Likert-type questions)**	
I understand how vaccines work.	
*Vaccines are safe.	
It is possible to have too many vaccinations.	
*I think vaccines should be a compulsory practice.	
I believe that vaccine-preventable diseases (like measles and mumps) can be serious.	
I feel a social pressure to be vaccinated or to vaccinate my children.	
My religious or cultural beliefs are compatible with the practice of vaccination.	
*My healthcare provider (for example my GP) has mine and/or my child’s best interests at heart.	
*I believe if I get vaccinated it would benefit the well-being of others.	
*Vaccines are a necessity for our health and well-being.	
News stories regarding vaccinations in the media have affected my views on this issue.	
I have already vaccinated or plan to vaccinate my children. (Leave blank if you are not planning to have/adopt children)	
**Section 3: Opinions on vaccinations (Yes/No questions with open-ended follow-up)**	
Do you think vaccinations have any risks?	
If YES, can you name any?	
Do you think vaccinations have any benefits?	
If YES, can you name any?	
Do you think the benefits associated with vaccinations outweigh the risks?	
Why?	
Do you believe there are other ways to prevent infectious diseases?	
If YES, which ones?	
Have the media impacted your views on the practice of vaccination?	
Why?	

### Survey distribution

The online survey was created using Google Forms and circulated by a group of six undergraduate students enrolled in the BSc(Hons) Biology and BSc(Hons) Biochemistry courses at the University of Portsmouth, UK. Due to the nature of the study, the volunteers were recruited via convenience sampling: the undergraduate investigators distributed the survey link between November 2019 and January 2020 within their network of contacts using various social media platforms, namely Facebook, Instagram, Twitter and email.

### Statistical analysis

Data were analysed using IBM SPSS Statistics 26. Given the ordinal nature of the data, Kruskal-Wallis nonparametric tests were used to compare the VCS between different groups, with a significance cut-off of *p* ≤ 0.05. When statistical significance was observed, post hoc analysis was carried out using pairwise Dunn’s tests, and the significance level adjusted for sample size via Bonferroni correction. Due to the small number of respondents in some of the demographic subgroups, participants’ responses were pooled with regard to ethnicity, religion and academic qualifications prior to the statistical analysis.

### Ethical approval

This study was conducted in accordance with the University of Portsmouth research ethics policy and guidelines. Ethical approval (BIOL-ETHICS#002–2019) was obtained from the School of Biological Sciences Ethics Representative prior to the start of the investigation. The online survey was prefaced by a disclaimer explaining the purpose of the study, its anonymous and voluntary nature, and the right to withdraw from it or leave questions unanswered. The participants were required to provide informed consent prior to accessing the questionnaire. All data were collected, handled and stored in accordance with the General Data Protection Regulation (GDPR).

## Results

The demographic characteristics of the 739 participants who completed the survey are presented in Table 2. With regard to age and gender, the study population is visibly skewed towards a demographic similar to that of the undergraduate students who circulated the survey: the majority of the participants were women (as were 5 out of 6 of the students who circulated the survey) in the 18–24 age range (the same as all of the students who circulated the survey). Given the experimental design of this study, this selection bias was not surprising: it reflects the well-documented human tendency to form homogeneous social groups with regard to gender and age, and hints that the study population is indeed representative of the students’ social sphere (Kiuru et al. 2009). The study population did not differ greatly from the general British population (as per the latest UK census carried out in 2011) with regard to ethnicity and, aside from a few discrepancies, religion (UK Office for National Statistics 2015).

**Table 2:** Demographic features of the study population (*n* = 739)

**Age**	**Count**	**%**	**Religion**	**Count**	**%**
18–24	336	45.5	Agnostic	110	14.9
25–30	84	11.4	Atheist	198	26.8
31–45	163	22.1	Buddhist	6	0.8
46–60	125	16.9	Christian	345	46.7
60+	31	4.2	Hindu	13	1.8
			Jewish	16	2.2
**Gender**	**Count**	**%**	Muslim	25	3.4
Female	590	79.8	Sikh	6	0.8
Male	145	19.6	Other	20	2.7
Nonbinary	3	0.4			
Other	1	0.1			
**Number of children**	**Count**	**%**	**Highest qualification**	**Count**	**%**
0	402	54.4	No qualification	5	0.7
1	97	13.1	Professional/vocational qualification	64	8.7
2	171	23.1	GCSE/O-Level	44	6.0
3	46	6.2	A-Level/BTEC	360	48.7
4 or more	23	3.1	Bachelor’s degree	181	24.5
			Postgraduate degree	79	10.7
			Other	6	0.8
**Ethnicity**				**Count**	**%**
Arab				4	0.5
Asian - Bangladeshi				2	0.3
Asian - Chinese				0	0.0
Asian - Indian				13	1.8
Asian - Other				15	2.0
Asian - Pakistani				3	0.4
Black - African				56	7.6
Black - Caribbean				17	2.3
Black - Other				4	0.5
Mixed - White and Asian				0	0.0
Mixed - White and Black African				1	0.1
Mixed - Other				9	1.2
Mixed - White and Black Caribbean				9	1.2
White - English/Welsh/Scottish/Northern Irish				552	74.7
White - Gypsy or traveller				0	0.0
White - Irish				10	1.4
White - Other				43	5.8
Other				1	0.1

*GCSE* General Certificate of Secondary Education, *O-Level* Ordinary level, *BTEC* Business and Technology Education Council

**Fig. 1:**
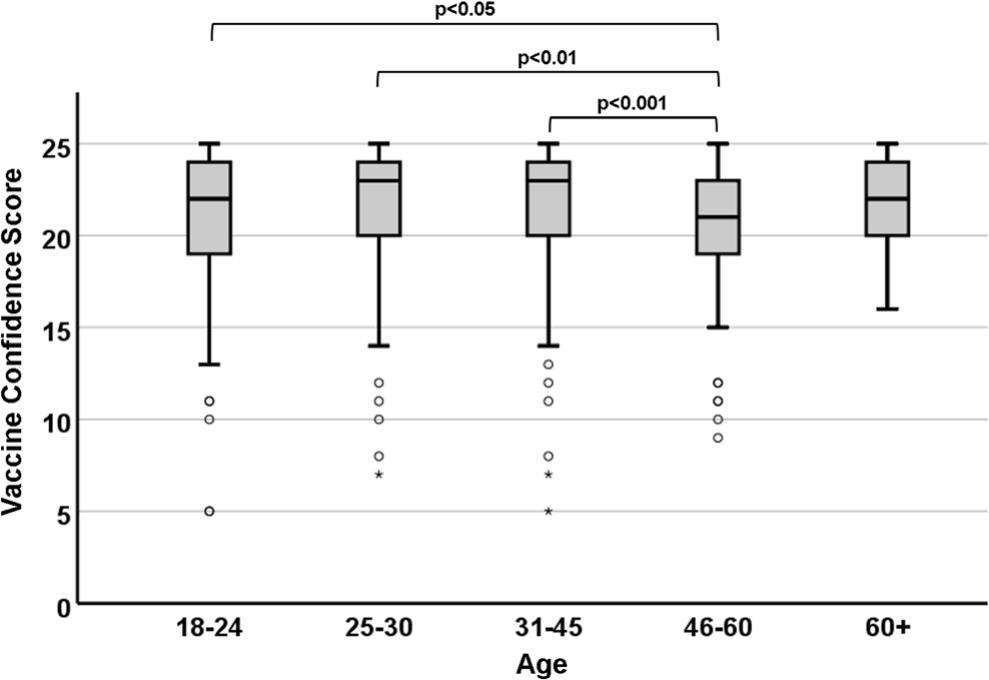
Association between participants’ age and their VCS. Participants in the 46–60-year age range showed significantly lower vaccine confidence compared to each of the younger groups, but not in comparison to older participants

As shown in Fig. 1, a significant difference (χ^2^ = 18.321; df = 4; *p* = 0.001) was observed in the median VCS among different age groups. Post hoc analysis identified that participants between 46 and 60 years old had the lowest VCS, with a statistically significant pairwise difference with regard to each of the other younger groups but not in comparison to the 60+ group. The difference was particularly accentuated (*p* = 0.001) between the groups aged 46–60 (median VCS = 21) and 31–45 years (median VCS = 23). A decreasing trend was observed with regard to age in the responses to “Have the media impacted your views on the practice of vaccination?”: participants in the 18–24 group were the most likely (23%) to give a positive answer, followed by 25–30 and 31–45 (both at 17%), 46–60 (16%) and 60+ (6.5%).

**Fig. 2.**
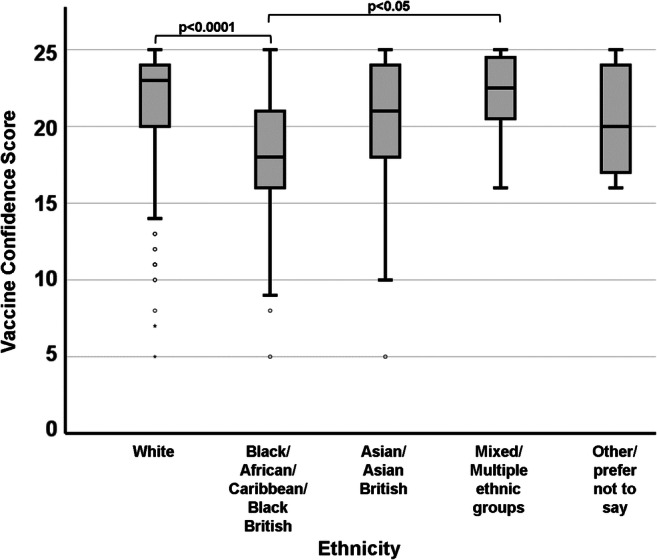
Association between participants’ ethnicity and their VCS. Participants from Black ethnic backgrounds showed significantly lower vaccine confidence than those from White or Mixed ethnicities

A strong significant difference (χ^2^ = 56.839; df = 4; *p* < 0.0001) in vaccine confidence was observed between participants belonging to different ethnicities (Fig. 2). It is important to note that although participants could indicate their exact background (e.g. Black - Caribbean) according to the UK government “list of ethnic groups” guidelines (GOV.UK list of ethnic groups 2020), all subgroups belonging to each individual category were pooled prior to the statistical analysis due to the very small number of participants belonging to certain subgroups. Participants from Black ethnic backgrounds (median VCS = 18) showed a significantly lower score compared to White (median VCS = 23; *p* < 0.0001) and Mixed (median VCS = 22.5; *p* = 0.015) backgrounds. No other statistically significant differences were observed with regard to ethnicity.

**Fig. 3.**
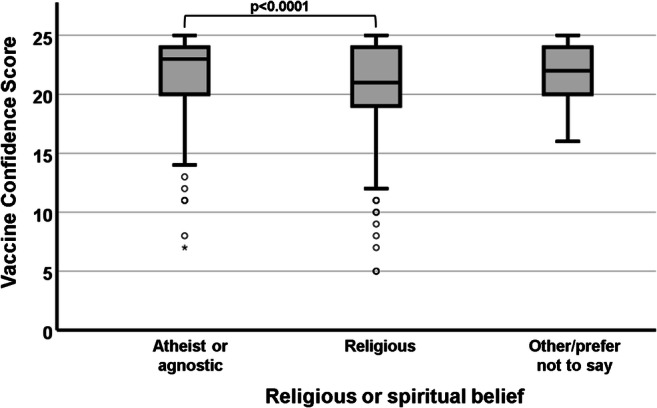
Association between participants’ religious or spiritual belief and their VCS. Non-religious participants showed significantly higher vaccine confidence than religious ones

Figure 3 highlights a strong difference (χ^2^ = 20.374; df = 2; p < 0.0001) in vaccine confidence between religious and non-religious respondents. As described in the paragraph above, participants were grouped into three broader categories (atheist/agnostic, religious, other/prefer not to say) due to the small number of responses recorded for some of the individual religions. Post hoc analysis indicated that religious participants (median VCS = 21) expressed significantly lower vaccine confidence than non-religious ones (median VCS = 23; *p* < 0.0001). Jewish respondents were the most likely (93.8%) to agree or strongly agree with the statement “My religious or cultural beliefs are compatible with the practice of vaccination”, followed by Buddhist (66.7%), Christian (56.5%), Muslim (52%), Sikh (50%) and Hindu (38.5%). However, unlike the aggregated values provided for religious versus nonreligious participants, these percentages should be considered mostly anecdotal due to the small number of participants in some of the subgroups.

**Fig. 4.**
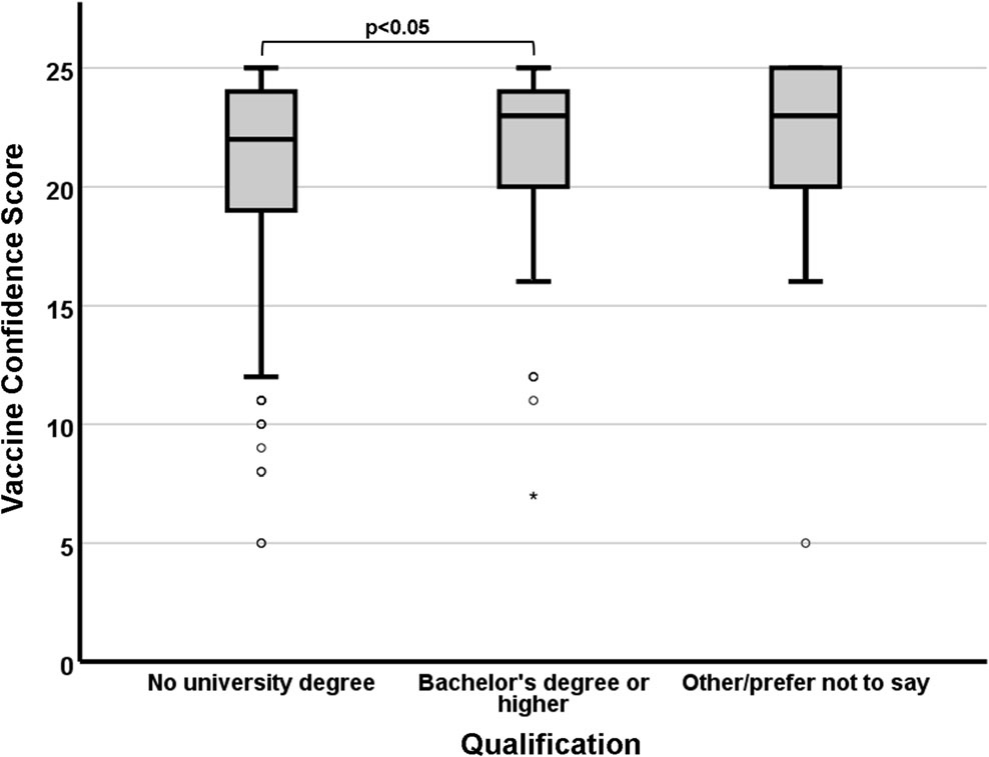
Association between participants’ academic qualifications and their VCS. Participants with a HE degree had significantly higher vaccine confidence than those who did not

The results shown in Fig. 4 indicate significant differences in median VCS (χ^2^ = 7.813; df = 2; *p* = 0.02) depending on their academic qualifications. As for the case of ethnicity and religion, participants’ qualifications were pooled into broader categories to account for the small number of responses recorded in some of the subgroups. Participants who achieved a university degree (undergraduate or higher) had a significantly higher (median VCS = 23; *p* = 0.017) median VCS than those who did not (median VCS = 22).

**Fig. 5.**
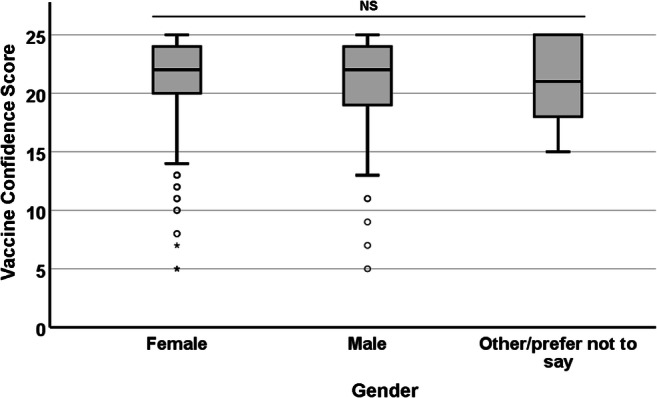
Association between participants’ gender and their VCS. None of the observed differences were statistically significant

**Fig. 6.**
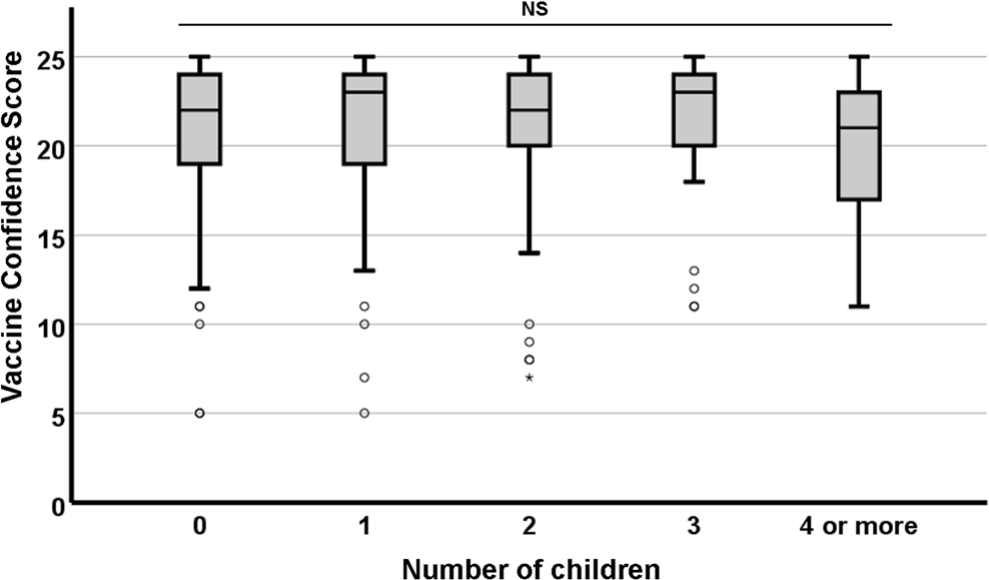
Association between participants’ parental status and their VCS. None of the observed differences were statistically significant

As shown respectively in Figs. 5 and 6, no significant differences in median VCS were observed with regard to gender (χ^2^ = 0.388; df = 2; *p* = 0.824) or parental status (χ^2^ = 3.833; df = 4; *p* = 0.422).

## Discussion

University students are a crucial demographic with regard to vaccination decision-making. First of all, for many of them the HE years (for the majority of UK students corresponding to the 18–24 age range) closely precede the average family planning and child-rearing period (Universities UK 2018). Furthermore, the university years represent the time period when many young adults become independent and responsible for their own health decision-making. Aside from their individual parenting and healthcare decisions, graduates play a fundamental role in shaping the society they live in by covering high responsibility roles (e.g. educators, policymakers, HCW, etc.) and influencing future generations. While issues related to immunisation and vaccine hesitancy are routinely taught in healthcare-related university courses (e.g. medicine, nursing, midwifery, pharmacy, etc.), they are understandably not a part of the vast majority of the other degree curricula (Vyas et al. 2018). As a result, after learning basic rudiments of immunology in school, most university students do not receive any further education on it, and are therefore susceptible to consulting unreliable or less authoritative sources such as the internet or their peer group to inform their opinion on this matter.

For the reasons discussed in the previous paragraph, it is of paramount importance to investigate the determinants of vaccine confidence (or lack thereof) in HE students. However, to the best of our knowledge, there is limited literature evaluating vaccine hesitancy in university students, and most existing studies discuss interventions carried out on the students themselves (particularly with regard to healthcare-related degrees) rather than focusing on their social sphere and on the factors that may influence their views and independent decision-making (Bralic and Pivalica 2019; Johnson et al. 2019; Vyas et al. 2018). Within the population surveyed in this study, vaccine confidence varied significantly with age, ethnicity, religion and graduate status of the participants, but not with their gender and number of children.

### Age

The present study unearthed a hitherto unreported association between age and vaccine hesitancy, whereby participants in the 46–60-year age range were significantly less vaccine-confident than those in any of the younger age groups. Interestingly, the Wellcome Monitor Report 2018 highlighted no effect of age on British citizens’ responses to three similar questions on vaccine confidence (NatCen Social Research 2019). While this discrepancy could be simply attributed to the different phrasing and number of questions used in the two surveys, it could allude to an effect specific to the demographic surveyed in our study, which would be “diluted” when collecting the opinions of the general population. A tentative explanation of the lower vaccine confidence of participants in the 46–60 age range, but not those over 60, could be suggested by considering that the average age of British first-time parents over the last two decades is approximately 30 years old (Office for National Statistics 2019). As a result, those currently in the 46–60-year age range are likely to have raised their first child and engaged in vaccination decision-making around the turn of the twenty-first century, in a period when Andrew Wakefield’s *Lancet* paper and the ensuing MMR controversy hit the media limelight, casting serious doubts on the safety of vaccinations. On the other hand, people that are currently over 60 years old are likely to have grown up in a period when most VPD were still perceived as a severe and concrete threat, and raised their children around the 1980s, prior to the MMR controversy and in correspondence to immunisation milestones such as the eradication of smallpox and near-eradication of other VPD such as polio and measles. This interpretation is further supported by the answers to the question “Have the media impacted your views on the practice of vaccination?”, whereby 16% of the participants in the 46–60 group gave a positive answer, compared to only 6.5% in the 60+ group.

### Ethnicity

Within the study population, we observed significant differences in vaccine confidence on the base of ethnicity, with participants from Black backgrounds expressing considerably lower confidence than those from White or Mixed backgrounds. It is important to remark that, as other key confounding factors such as economic status and geographical location were not investigated in the present study, no conclusions should be drawn as to the existence of a causative link between the variables in object. A clear example of the non-causative nature of the association between ethnicity and vaccine hesitancy is provided by the Wellcome Global Monitor 2018 report: vaccine confidence is considerably higher in several developing countries with a predominantly Black population (e.g. Ethiopia, Burundi, Rwanda, Sierra Leone, etc.) than in developed countries with a predominantly White population (e.g. France, Austria, Russia) and amongst Black communities living in developed countries (Wellcome Trust 2019). These observations reinforce the notion that the factors determining vaccine hesitancy are likely to be environmental (i.e. economic, social, cultural) rather than ethnical. While our results corroborate evidence described in a recent systematic review discussing the lower vaccine uptake in Black and Asian Minority Ethnic (BAME) groups in the UK with regard to the perspectives of participants from Black backgrounds, they did not confirm previous observations of increased hesitancy amongst those from Asian backgrounds (Forster et al. 2017).

### Religion

Our results show that participants who identified themselves as religious had a significantly lower VCS than their atheist or agnostic counterparts. Although most religions do not explicitly forbid followers from receiving vaccinations, and in fact several religious authorities are vocal advocates of the practice of immunisation, there is no shortage of reports of vaccinations being refused on religious grounds (Imdad et al. 2013; Pelčić et al. 2016; Ruijs et al. 2012). While the survey highlighted a variability in participants’ perceived compatibility of their specific creed with the practice of vaccination, a larger sample number would be advisable in order to pinpoint the extent of vaccine confidence amongst individual religions. Further research would also be required to clarify the intersectionality of religion with other factors (e.g. gender, ethnicity, socioeconomic status) that might influence vaccine hesitancy in a creed-specific manner.

### Academic qualifications

Academic qualifications had previously been recognised as predictors of vaccine confidence, with highly educated parents being more aware of the risks posed by VPD and therefore more likely to vaccinate their children (Lee and Sibley 2020; Luman et al. 2003). However, these observations are in apparent contradiction to other reports that indicate that academically qualified people can be less inclined to trust policymakers and HCW and therefore research independent sources of information such as the internet or their peer group, often adopting or promoting conspiratorial or anti-vaccination standpoints (Patel and Berenson 2013). By showing that participants with a HE degree have significantly higher vaccine confidence than those without one, our results corroborate the former hypothesis, i.e. that a lower educational status may be a contributing factor to (or at least an indicator of) vaccine hesitancy.

## Conclusions

The ongoing Covid-19 pandemic provides a sobering glimpse of the dramatic impact of transmissible diseases in the absence of a suitable vaccine, reinforcing the vital necessity of maintaining vaccine coverage above the herd immunity threshold at both the global and local level to prevent the re-emergence of serious VPD (Harrison and Wu 2020). University students are a key demographic to be considered when developing and implementing short- and medium-term strategies aimed at tackling vaccine hesitancy in current and prospective parents. Moreover, graduates play a fundamental role in shaping our society and forming tomorrow’s citizens, making them ideal candidates to foster scientific literacy and increase long-term vaccine acceptance.

The present study provides novel insight into the factors underpinning vaccine hesitancy in the social sphere of undergraduate students. In particular, middle-age has been identified as a critical period due to its strong association with vaccine hesitancy. This specific age range presumably corresponds in no small part to students’ parents or older relatives and their acquaintances, which represent a potential source of influence on their decision-making with regard to child-rearing and vaccination. Within the study population, participants from Black ethnic backgrounds were significantly less vaccine-confident, highlighting the importance of further investigations to elucidate the social, economic and cultural factors that may specifically affect vaccine confidence amongst Black background communities. Similarly, the observation of high levels of vaccine hesitancy amongst religious participants reinforces the necessity of involving religious leaders at both the national and local level to minimise the likelihood of vaccinations being refused on religious grounds. By showing a strong positive association between possession of a HE degree and vaccine confidence, our results provide further clarity on the relationship between vaccine confidence and academic qualifications, supporting previous observations that lower educational status is linked to vaccine hesitancy.

While instrumental to the experimental approach of this study, the non-random sampling strategy used in the survey does carry inherent limitations. In particular, the fact that the survey was distributed by Biology and Biochemistry undergraduate students may mean that participants could have a better understanding of biomedical matters such as the importance of vaccinations. Together with the fact that people with a science background may feel more inclined to participate in this type of survey, this could potentially introduce an element of selection bias that is important to be aware of.

Another limitation of the present study is the small number of participants belonging to certain demographic subgroups; further research would be advisable to precisely pinpoint the extent of vaccine hesitancy amongst each individual subgroup. Moreover, while the study evaluated the impact on vaccine hesitancy of six key demographic factors, future studies could investigate the combined effect of their interactions from an intersectional perspective. In this sense, the Vaccine Confidence Score developed in this study has proven to be a powerful tool to gauge subtle differences that would be overlooked when separately analysing answers to individual questions, and could easily be implemented or adapted by researchers, HCW and policymakers wishing to quantitatively investigate vaccine hesitancy within a target population.
